# Supplementary material for: Stacking Model‐Based Classifiers for Dealing With Multiple Sets of Noisy Labels
Source: Biom J. 2025 Mar 12;67(2):e70042. doi: 10.1002/bimj.70042 (PMC11898607; doi:10.1002/bimj.70042)
Supplement: Supplementary file 1 — Supporting Information [file BIMJ-67-e70042-s001.zip › density-based-ensemble-model-main/competitors_real_data/netlab-master/netlab-master/help/gradchek.htm]

Netlab Reference Manual gradchek


# gradchek

## Purpose

Checks a user-defined gradient function using finite differences.

## Synopsis

```
gradchek(w, func, grad)
[gradient, delta] = gradchek(w, func, grad)
gradchek(w, func, grad, p1, p2, ...)
```

## Description

This function is intended as a utility for other netlab functions
(particularly optimisation functions) to use. It enables the user
to check whether a gradient calculation has been correctly implmented
for a given function.
`gradchek(w, func, grad)` checks how accurate the gradient
`grad` of a function `func` is at a parameter vector `x`.
A central
difference formula with step size 1.0e-6 is used, and the results
for both gradient function and finite difference approximation are
printed.
The optional return value `gradient` is the gradient calculated
using the function `grad` and the return value `delta` is the
difference between the functional and finite difference methods of
calculating the graident.

`gradchek(x, func, grad, p1, p2, ...)` allows additional arguments
to be passed to `func` and `grad`.

## See Also

`conjgrad`, `graddesc`, `hmc`, `olgd`, `quasinew`, `scg`

---

**Pages:**
Index


---

Copyright (c) Ian T Nabney (1996-9)
